# Effects of CO_2_ Modified Atmosphere Packaging on Lipid Oxidation, Antioxidant Capacity and Transcriptome Responses of Fresh Edible Peanuts

**DOI:** 10.3390/foods15173042

**Published:** 2026-08-28

**Authors:** Yilin Huang, Yuxin Xiu, Ruilu Liu, Wenchao Liu, Linlin Li, Tongxiang Yang, Weiwei Cao, Haomeng He, Guangfei Zhu, Shanshan Tie, Guangyue Ren, Xu Duan, Libo Wang

**Affiliations:** 1 College of Food and Bioengineering, Henan University of Science and Technology, Luoyang 471023, China; thriving11@163.com (Y.X.); 13849176696@163.com (R.L.); wen_chaoliu@haust.edu.cn (W.L.); hustlinlinli@126.com (L.L.); txyamy@163.com (T.Y.); weiweihappy2@163.com (W.C.); hehaomeng@haust.edu.cn (H.H.); tieshanshan@haust.edu.cn (S.T.); rgy@haust.edu.cn (G.R.); duanxu_dx@163.com (X.D.); 2 Academy of Agricultural Planning and Engineering, Ministry of Agriculture and Rural Affairs, Haidian, Beijing 100125, China; zhuguangfei@aape.org.cn

**Keywords:** peanuts, CO_2_-MAP, lipid oxidation, antioxidant capacity, transcriptome analysis

## Abstract

Understanding the effect of carbon dioxide (CO_2_) modified atmosphere packaging (MAP) on postharvest quality and its molecular mechanism in peanuts will be helpful for the application and optimization of CO_2_-MAP. The appearance quality, lipid oxidation indices (acid value, carbonyl value, and malondialdehyde content), DPPH free radical scavenging rate, and the activity of superoxide dismutase (SOD) and lipoxygenase (LOX) of fresh edible peanuts packaged with CO_2_ or air (CK, control group) were determined at 7 d intervals for 35 d. Transcriptome sequencing (RNA-seq) of peanuts was performed to identify differentially expressed genes (DEGs) and enriched functional pathways. CO_2_-MAP could maintain the appearance quality of peanut pods and kernels. Compared with the control, CO_2_-MAP maintained a lower acid value (14.3% lower), carbonyl value (10.7% lower), and malondialdehyde content (22.5% lower), suppressed LOX activity (23.4% lower), as well as retained higher DPPH radical scavenging activity (31.1% higher) and SOD activity (25.0% higher) at 35 d. Transcriptomics identified 3575 downregulated and 2166 upregulated DEGs between the CO_2_-MAP and CK. These DEGs were significantly enriched in pathways related to energy metabolism, carbon metabolism, lipid metabolism, signal transduction, and antioxidant functions. Collectively, CO_2_-MAP effectively delayed quality deterioration of peanuts by suppressing lipid oxidation, enhancing antioxidant capacity, reprogramming energy metabolism, and attenuating senescence-associated signaling pathways.

## 1. Introduction

Peanut (*Arachis hypogaea* L.) is one of the most important oilseed and economic crops all over the world, and widely appreciated for its outstanding nutritional value and high oil content [1,2,3]. Peanut kernels are rich in protein, unsaturated fatty acids, vitamins, minerals, and contain various bioactive compounds such as phenolics, flavonoids, and ascorbic acid [4,5]. Fresh edible peanuts refer to the freshly peanuts eaten directly (raw peanuts) or boiled and eaten (boiled peanuts) after harvest, and have gained growing consumer popularity owing to their delicious taste, convenience of consumption, and maximal retention of bioactive nutrients, etc. [6,7]. As living standards improve, people are placing greater emphasis on the quality of food, including nutrition and flavor, leading to a growing demand for fresh-eaten peanuts [8].

However, the high moisture and lipid content of fresh peanuts result in highly susceptible to quality deterioration [9], and are compounded by the risk of mold spoilage caused by microbial colonization on pod surfaces. Post-ripening processes trigger the release of heat and moisture, as well as a cascade of lipid oxidation reactions. In addition, fresh peanuts are abundant in polyunsaturated fatty acids [10], and their inherent double-bond structures confer extreme susceptibility to oxidative degradation [11]. These factors lead to high susceptibility to mold, quality deterioration, oil rancidity, and poor storage stability of fresh edible peanuts after harvest [12], which restricts the development of the fresh edible peanut industry. Therefore, developing efficient, eco-friendly, and safe postharvest preservation technologies and clarifying the underlying molecular regulatory mechanisms are of great significance for promoting the high-quality development of the fresh peanut industry.

Modified atmosphere packaging (MAP) is one of the most successful storage techniques that can effectively alleviate quality deterioration of agricultural and food products [13], satisfying consumers’ comprehensive demands for food safety, nutritional integrity, and flavor retention. The use of MAP has been used for the preservation of a wide variety of agricultural and food products, such as grains, fruits, vegetables, dried fruits, and nuts [14,15]. CO_2_ filling, N_2_ filling, and vacuuming were three common MAP storage methods to provide low-oxygen atmospheres. CO_2_-MAP has been proved to be effective in extending the post-harvest longevity of fruit and vegetables [16,17], delaying senescence and extending the storage period of edible fungus [18], and prolonging the postharvest longevity of cut peonies [19]. However, its operation and maintenance costs are high, so it is only widely used in bulk agricultural products [19].

For peanuts, multiple studies have demonstrated that hermetic storage could limit fungal growth and the accumulation of aflatoxins [20,21,22]. The oxygen level is a major factor that could affect the deterioration and oxidation of peanuts, and the quality deterioration and oxidation product accumulation could be greatly suppressed under low oxygen atmospheres in storage or transportation [23]. Meanwhile, the dried peanut pods with MAP storage (vacuum packaged) showed higher oil and protein content and lower change in moisture content, free fatty acid, aflatoxin content, physiological loss in weight, and color change after 180 d of storage [24]. Nitrogen (N_2_) MAP treatment effectively preserved commercial quality of fresh edible peanuts by restraining browning, maintaining firmness, and delaying the accumulation of malondialdehyde and hydrogen peroxidase, the production of superoxide anion, and the electrolyte leakage rate [25]. CO_2_-MAP inhibited mycobiota development and conserved the color and lipid oxidation quality of dried peanut kernels (6.4% moisture content) for at least 15 weeks [26]. Compared with hermetic sealing, 99% CO_2_-MAP achieved superior quality preservation for peanuts with 7.0% and 8.0% moisture contents [27]. By simultaneously lowering the ambient oxygen (O_2_) partial pressure and elevating CO_2_ levels, MAP storage suppressed respiratory metabolism, curtails microbial proliferation, and retards tissue softening [19]. Notably, peanuts are capable of adsorbing CO_2_, and the peanuts after CO_2_ adsorption could impede the inward diffusion of O_2_. Consequently, peanuts sealed in packages with CO_2_ sustained lower internal O_2_ concentrations and thereby mitigated oxidative rancidity and quality deterioration [28]. Previous MAP research on peanuts mainly focused on dried peanut kernels with low moisture (lower than 10%) or only physiological index detection. Although these studies show the potential advantage of MAP storage for maintaining peanut seed quality, the occasional negative effects call for a better understanding of the physiological and biochemical processes that occur during storage. Systematic studies on appearance quality, lipid oxidation, antioxidant capacity, enzyme activity, and transcriptome sequencing to resolve underlying molecular regulatory mechanisms of fresh edible peanuts under CO_2_-MAP are still lacking and need to be comprehensively elucidated.

The changes in O_2_ and CO_2_ levels in the storage environment may affect the oxidation degree, metabolism pathway, and gene expression of peanuts, which can be investigated by transcriptomics. Transcriptome sequencing (RNA-seq) is widely used to investigate the genotypic phenotype, which could identify changes in gene expression and key pathways [29]. Hence, transcriptome sequencing could be used to reveal the molecular mechanism of quality maintenance for CO_2_-MAP on fresh edible peanut. In the present study, the effect of CO_2_-MAP on the appearance quality, lipid oxidation indices, antioxidant capacity, enzyme activity, and molecular mechanism of fresh edible peanuts (dry shells and wet kernels) at different storage durations was investigated. This study aims to reveal the quality changes of fresh edible peanuts and their molecular mechanism, and will provide a deep understanding of the molecular mechanism and theoretical basis for the application of fresh edible peanut storage technology.

## 2. Materials and Methods

### 2.1. Fresh Edible Peanut Treatments

The fresh edible peanut (Yuhua 93) was harvested from a peanut farm (Puyang, Henan Province, China). Fresh peanut pods with uniform maturity, consistent size, free of mechanical damage and insect/disease infestation were manually picked for the test. Surface soil adhered to the pods was rinsed away with running water, and then the pods were air-dried at room temperature to eliminate visible moisture on the shell surface.

Based on the moisture changes of peanut kernels, peanut shells, and whole pods in our preliminary hot air-drying experiment, the peanut pods were subjected to moderate drying at 45 °C for 4 h. Under this moderate drying condition, the drying efficiency was high, and the peanuts reached the state of “dry shells and wet kernels (Moisture content decreased from the initial 62% to 21% of peanut shells and decreased from the initial 45% to 33% of peanut kernels)”. This treatment could effectively inhibit the growth and reproduction of microorganisms, while maximizing the freshness of the fresh edible peanut kernels.

### 2.2. MAP Treatment with CO_2_

After moderate drying, peanut pods were randomly divided into 200 g samples and enclosed in the packaging bags of polyamide/polyethylene (PA/PE, The PA layer thickness is 0.048 mm, and the PE layer thickness is 0.192 mm.) composite film (0.24 mm thickness, 25 cm × 35 cm), which were purchased from Hebei Hengwang Plastic Industry Co., Ltd. (Xingtai, China). The oxygen transmission rate of packaging bags was 48.7 cm^3^/(m^2^·24 h·0.1 MPa), and the water vapor transmission rate was 3.13 g/(m^2^·24 h). The parameters are provided by the manufacturer through testing.

For the CO_2_-MA group, each bag was vacuumized for 20 s, then filled with high-purity CO_2_ (≥99.999%; supplied by Taishan Fire Equipment Supply Station, Luoyang, Henan Province, China) at a pressure of 0.03 MPa for 10 s prior to heat sealing. The control group was performed using the same procedure but was filled with air instead of CO_2_. All packaged samples were stored in the dark in a constant-temperature incubator at 35 °C for 35 d, and random sampling was performed every 7 d.

### 2.3. The Appearance of Peanut Pods and Peanut Kernels

The pictures were taken under a fixed illumination source (daylight fluorescent lamp with color temperature of 3500 K) using a camera at a vertical distance of 10 cm from the peanut samples [25].

### 2.4. Determination of Lipid Oxidation Indicators

#### 2.4.1. Peanut Oil Extraction

Peanut oil was extracted from the peanut kernel granules with petroleum ether as described in Qu et al. [30]. The peanut kernels were sliced into approximately 1 mm thick pieces, then the peanut thin slices were ground into powders. Petroleum ether was added at twice the volume of the kernel samples, and the mixture was homogenized, followed by 12 h of maceration. The leachate was filtered through filter paper layered with anhydrous sodium sulfate, and the filtrate was concentrated via vacuum rotary evaporation at 40 °C under 0.09–0.10 MPa. The resulting oil residue was collected and used for further chemical analysis.

#### 2.4.2. Acid Value

The acid value (AV) was determined by the method described by Qu et al. [31] with modifications. The extracted oil sample was placed into a conical flask and dissolved in 100 mL ether-isopropanol solution (anhydrous ether and isopropanol were mixed evenly in a volume ratio of 1:1). Then the solution was titrated with standard KOH solution (0.1 mol/L) with phenolphthalein as the indicator until the solution turned slightly red and did not fade within 15 s. Each treatment comprised three biological duplicates and three technical duplicates for each biological duplicate in the determination of lipid oxidation indicators. The acid value was calculated as follows:

(1) $$AV(mg/g)=\frac{\left(V-V_{0}\right)\times c\times 56.1}{m}$$

where AV is the acid value of the sample (mg/g); V is the volume (mL) of the standard solution consumed by the titration sample; V_0_ is the volume (mL) consumed in the blank test; c is the concentration (mol/L) of the potassium hydroxide standard solution; 56.1 is the molar mass (g/mol) of potassium hydroxide; m is the mass (g) of the oil sample.

#### 2.4.3. Carbonyl Value

The carbonyl value (CV) was analyzed as described in Liu et al. [32] and Liu et al. [33]. The peanut oil sample (0.2 g) was dissolved in 5 mL benzene in a tube, and then each sample was added to 3 mL of trichloroacetic acid solution and 5 mL of 2,4-dinitrophenyl hydrazine. After mixing well, the mixture was incubated in a 60 °C water bath for 30 min and cooled to room temperature. 10 mL potassium hydroxide-methanol solution was slowly added along the tube wall, followed by vortex mixing and standing for 10 min. The absorbance of the mixture was measured at 440 nm. The carbonyl value was calculated as follows:

(2) $$CV(meq/kg)=\frac{A}{854\times m}\times 1000$$

where CV is the carbonyl value of the sample (meq/kg); A is the absorbance of the sample solution during the measurement; m is the mass of the oil sample (g); 1000 is the conversion factor.

#### 2.4.4. Malondialdehyde (MDA) Content

The content of MDA was measured according to the method reported by Li et al. [34] and Wu et al. [25] with modifications. Briefly, 1 g of peanut kernel powder was mixed with 5 mL of trichloroacetic acid solution in a stoppered Erlenmeyer flask. The mixture was centrifuged for 15 min at 4 °C and 8000× *g*. Then, 2 mL of the supernatant and 2 mL of thiobarbituric acid were mixed and boiled for 20 min. After cooling, the mixture was centrifuged for 15 min at 4 °C and 12,000× *g*, and the supernatant was collected for measurement. The absorbance was measured at 532, 450, and 600 nm. The malondialdehyde content was calculated as follows:

(3) $$X(mg/kg)=\frac{c\times V\times 1000}{m\times 1000}$$

where X is the content of malondialdehyde in the sample (mg/kg); c is the concentration of the sample solution (µg/mL); V is the volume of the sample (mL). m is the mass of the sample (g); 1000 is the conversion factor.

### 2.5. DPPH Free Radical Scavenging Activity

The DPPH free radical scavenging rate was conducted using a commercial DPPH free radical scavenging capacity assay kit (A153-1-1, Nanjing Jiancheng Bioengineering Institute, Nanjing, Jiangsu, China). 0.1 g of the peanut kernel powder was mixed with 1 mL of 80% methanol solution and homogenized in an ice-water bath. The mixture was centrifuged at 12,000 r/min for 10 min, and the supernatant was kept on ice for measurement. The detailed procedure was carefully followed according to the instructions provided by the manufacturer. 400 μL of sample was mixed with 600 μL reagent in the kit (sample test) or absolute ethyl alcohol (control), and 400 μL 80% methanol was mixed with 600 μL reagent in the kit (blank). After being placed in the dark at 25 °C for 30 min, the mixtures were centrifuged at 4000 r/min for 5 min. The absorbance value was measured at 517 nm. Each treatment comprised three biological duplicates and three technical duplicates for each biological duplicate. The DPPH free radical scavenging rate was calculated as follows:

(4) $$X\;\left(\%\right)=\left(1-\frac{A_{sample}-A_{control}}{A_{blank}}\right)\times 100\%$$

where X is the DPPH free radical scavenging rate (%); A_sample_ is the absorbance of the measurement group; A_control_ is the absorbance of the control group; A_blank_ is the absorbance of the blank group.

### 2.6. Enzyme Activity

#### 2.6.1. Superoxide Dismutase (SOD) Activity

SOD activity was determined using a superoxide dismutase assay kit (A001-3, Nanjing Jiancheng Bioengineering Institute, Nanjing, Jiangsu, China). 0.1 g of the peanut kernel powder was homogenized with 1 mL pre-chilled phosphate buffer (0.1 mol/L, pH 7.0–7.4), and centrifuged at 3000 r/min for 10 min. The supernatant was collected for further assay. The assay was performed in 96-well plates, including a control group (20 μL distilled water and 20 μL enzyme solution), blank control group (20 μL distilled water and 20 μL enzyme dilution solution), test group (20 μL sample solution and 20 μL enzyme solution), and blank group (20 μL sample solution and 20 μL enzyme dilution solution). 200 μL of substrate application solution was added to every well. After gentle mixing, the plate was incubated at 37 °C for 20 min, and absorbance was detected at 450 nm with a microplate reader. Each treatment comprised three biological duplicates and three technical duplicates for each biological duplicate in the determination of enzyme activity. The activity of SOD was calculated as follows:

(5) $$\mathrm{SOD}\;activity\left(U/g\right)=\frac{\mathrm{SOD}\;\mathrm{inhibition}\;\mathrm{percentage}\;\left(\%\right)}{50\%}\times \frac{V_{\mathrm{total}\;}}{V_{sample}}\times N÷\frac{W}{V_{total\;sample}}$$

where V_total_ is the total volume of the reaction system (mL); V_sample_ is the sample loading volume during measurement (mL); N is the dilution factor applied to the sample prior to testing; W is the mass of the sample being tested (g); V_total sample_ is the total volume of homogenization medium added during sample preparation (mL). The SOD inhibition percentage (%) was calculated as follows:

(6) $$\mathrm{SOD}\;\mathrm{inhibition}\;\mathrm{percentage}\;\left(\%\right)=\left(1-\frac{A_{\mathrm{measurement}}-A_{\mathrm{measurement}\;\mathrm{blank}}}{A_{control}-A_{control\;\mathrm{blank}}}\right)\times 100\%$$

where A_measurement_, A_measurement blank_, A_control_, and A_control blank_ represent the absorbance values of the measurement well, the measurement blank well, the control well and the control blank well, respectively.

#### 2.6.2. Lipoxygenase (LOX) Activity

LOX activity was determined using a lipoxygenase assay kit (Beijing Boxsin Technology Co., Ltd., Beijing, China). 0.1 g of the peanut kernel powder was homogenized with 1 mL extraction buffer in an ice bath. The homogenate was centrifuged at 15,000 r/min for 20 min at 4 °C, and the supernatant was collected as the crude enzyme solution and kept on ice for analysis. The assay group was supplemented sequentially with 100 µL crude enzyme solution, 800 µL Reagent I, and 100 µL Reagent II in a 1 mL quartz cuvette. For the blank group, 100 μL distilled water replaced the crude enzyme solution, while the other reagents remained identical to those of the assay group. After mixing, timing was started immediately. The absorbance at 234 nm was measured at total reaction times of 15 s and 75 s, denoted as A_1_ and A_2_, respectively. The activity of LOX was calculated as follows:

(7) $$LOX\;activity\left(U/g\right)=10000\times \frac{\left(A_{2m}-A_{1m}\right)-(A_{2b}-A_{1b})}{W}$$

where A_2m_, A_1m_, A_2b_, and A_1b_ represent the absorbance values of the sample at 75 s, sample at 15 s, blank at 75 s, and blank at 15 s, respectively; W is the mass of the sample (g).

### 2.7. RNA Isolation and Transcriptomic Sequencing

The peanut seed samples were flash-frozen with liquid nitrogen and stored in a −80 °C refrigerator for subsequent RNA extraction and transcriptomic sequencing. Total RNA was extracted from peanut seed samples using Trizol reagent (Beijing Solaibao Technology Co., Ltd., Beijing, China) following the manufacturer’s protocols. RNA was quantified using a NanoDrop spectrophotometer (BioPhotometer D30, Eppendorf AG, Hamburg, Germany). An OD260/OD280 ratio within the range of 1.8–2.0 indicated that the extracted RNA was of high purity, and the samples were therefore deemed acceptable. The integrity of total RNA was measured using an Agilent 2100 Bioanalyzer system (Agilent Technologies (China) Co., Ltd., Beijing, China). Subsequently, the qualified RNA was sent to the company (Qingke Biotechnology Co., Ltd, Beijing, China) for transcriptomic sequencing. Each treatment comprised three biological duplicates and three technical duplicates for each biological duplicate.

RNA purification, reverse transcription, library construction, and sequencing were performed at Qingke Biotechnology Co., Ltd. RNA libraries were constructed using the Hieff NGS^®^ mRNA Isolation Master Kit (Yeasen Biotechnology Co., Ltd., Shanghai, China), following the standard protocol for library construction and quality control. Quantitative quality control was performed using Qubit (Thermo Fisher Scientific Inc., Shanghai, China), and the sequencing analysis was performed with the BGI DNBSEQ-T7 gene sequencer (BGI Genomics Co., Ltd., Shenzhen, China).

First, reads with low quality, contaminated joints, and high content of unknown base (N) were filtered, and a quality assessment was carried out to ensure the reliability of the results. Trinity software (Trinity-2.15.1) was used to perform de novo assembly of the clean reads. The assembled transcripts were then clustered and deduplicated using Tgicl software (Tgicl-2.1) to obtain unigenes. Gene function was annotated based on the following databases: Nr (NCBI non-redundant protein sequences); Nt (NCBI non-redundant nucleotide sequences); Pfam (Protein family); KOG/COG (Clusters of Orthologous Groups of proteins); Swiss-Prot (A manually annotated and reviewed protein sequence database); KO (KEGG Ortholog database); GO (Gene Ontology). Gene expression was normalized by the FPKM (Fragments Per Kilobase of transcript per Million mapped reads) method. Differential expression analysis of two groups was performed using the DESeq2 R package (1.26.0). Genes with FDR < 0.05 & |log2 (foldchange)| ≥ 1 found by DESeq2 were assigned as differentially expressed. Differentially expressed genes (DEGs) were classified by function and biological pathway based on Gene Ontology (GO) and Kyoto Encyclopedia of Genes and Genomes (KEGG) annotation results and official classifications, and enrichment analysis was performed using the phyper function in R (R-4.52.2). The sequencing data were submitted to the NCBI Database (BioProject accessions: PRJNA1502420).

### 2.8. Statistical Analysis

Data were analyzed using IBM SPSS Statistics Version 20 software. Results were presented as the means ± SD (standard deviation). Statistical analysis was carried out using one-way analysis of variance (ANOVA), and statistical significance was determined by Tukey’s test at the 0.05 level.

## 3. Results

### 3.1. Changes in the Appearance of Peanut Pods and Peanut Kernels

**Figure 1:**
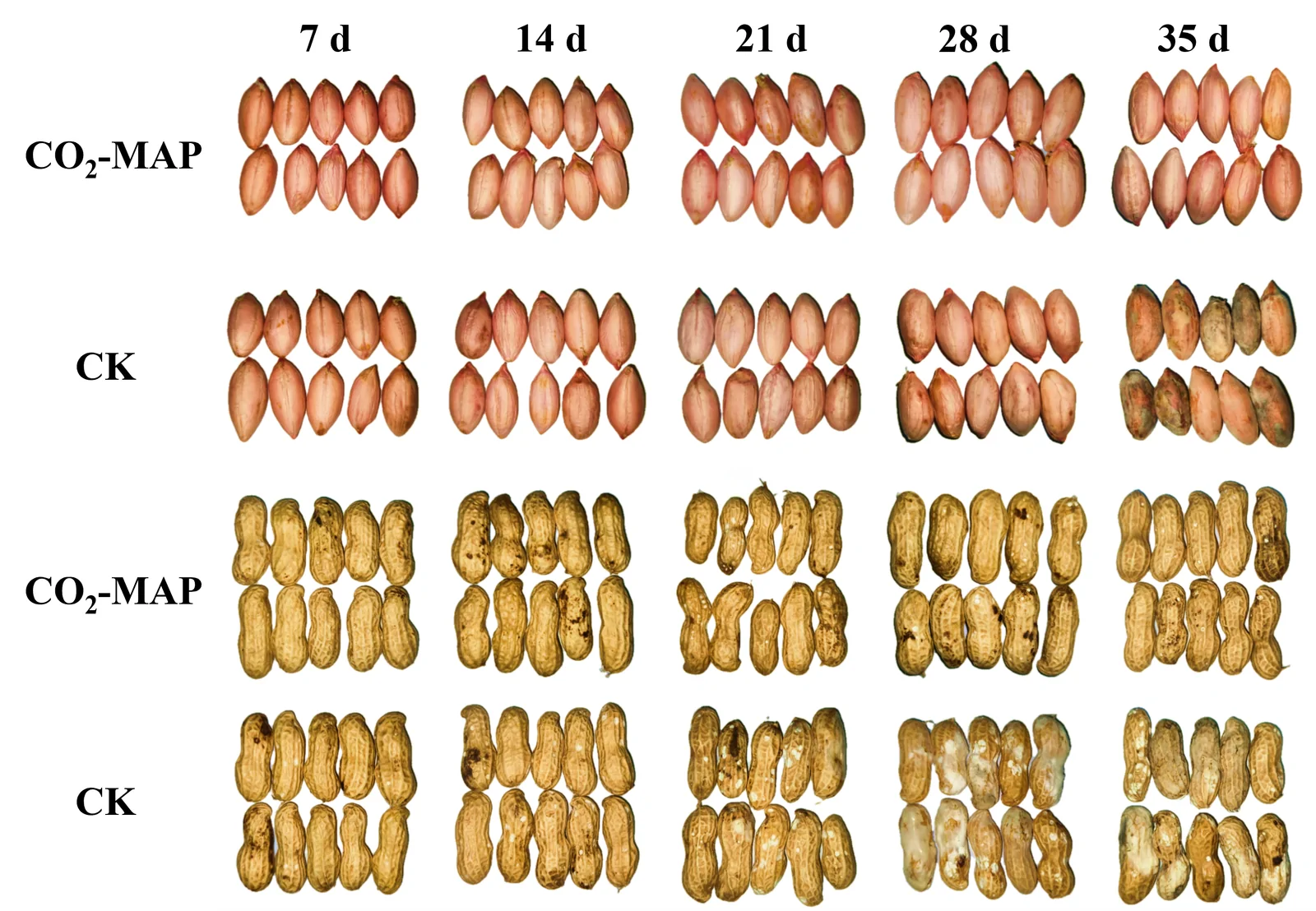
Changes in the appearance of fresh edible peanut pods and kernels at different storage times.

CO_2_-MAP treatment significantly maintained the color and delayed mold growth of peanuts during storage (Figure 1). This effect emerged as early as 7 d of storage and became particularly pronounced after 21 d. Peanut kernels in the CO_2_-MAP group maintained a uniform light pink color, plump texture, and smooth surface throughout the 35 d storage period, with no obvious shrinkage, visible browning symptoms, or mold growth. Only a very slight darkening of color was observed at 35 d, and the overall appearance remained close to the fresh kernels. In contrast, the appearance deterioration of peanut kernels in the control group intensified significantly with prolonged storage. Localized visible browning and slight shrinkage appeared at 21 d, and the browning area expanded continuously after 28 d, accompanied by a sunken, dehydrated surface on some kernels. At 35 d, most kernels exhibited dark brown color and visible mold spots, resulting in a drastic decline in commercial appearance quality.

For in-shell peanuts, the CO_2_-MAP group maintained a uniform light-yellow shell color with firm and intact structure throughout storage, and only a very slight color darkening and mold spots were observed after 21 d. In contrast, peanuts in the CK group deteriorated at a much faster rate. Localized dark and mold spots appeared at 7 d, and mold growth accelerated sharply with storage time. After 21 d, the shell color turned dark brown with visible white/green mycelium, and the structure became loose and fragile, leading to a severe loss of appearance quality.

### 3.2. Changes in Acid Value, Carbonyl Value, and MDA Content

**Figure 2:**
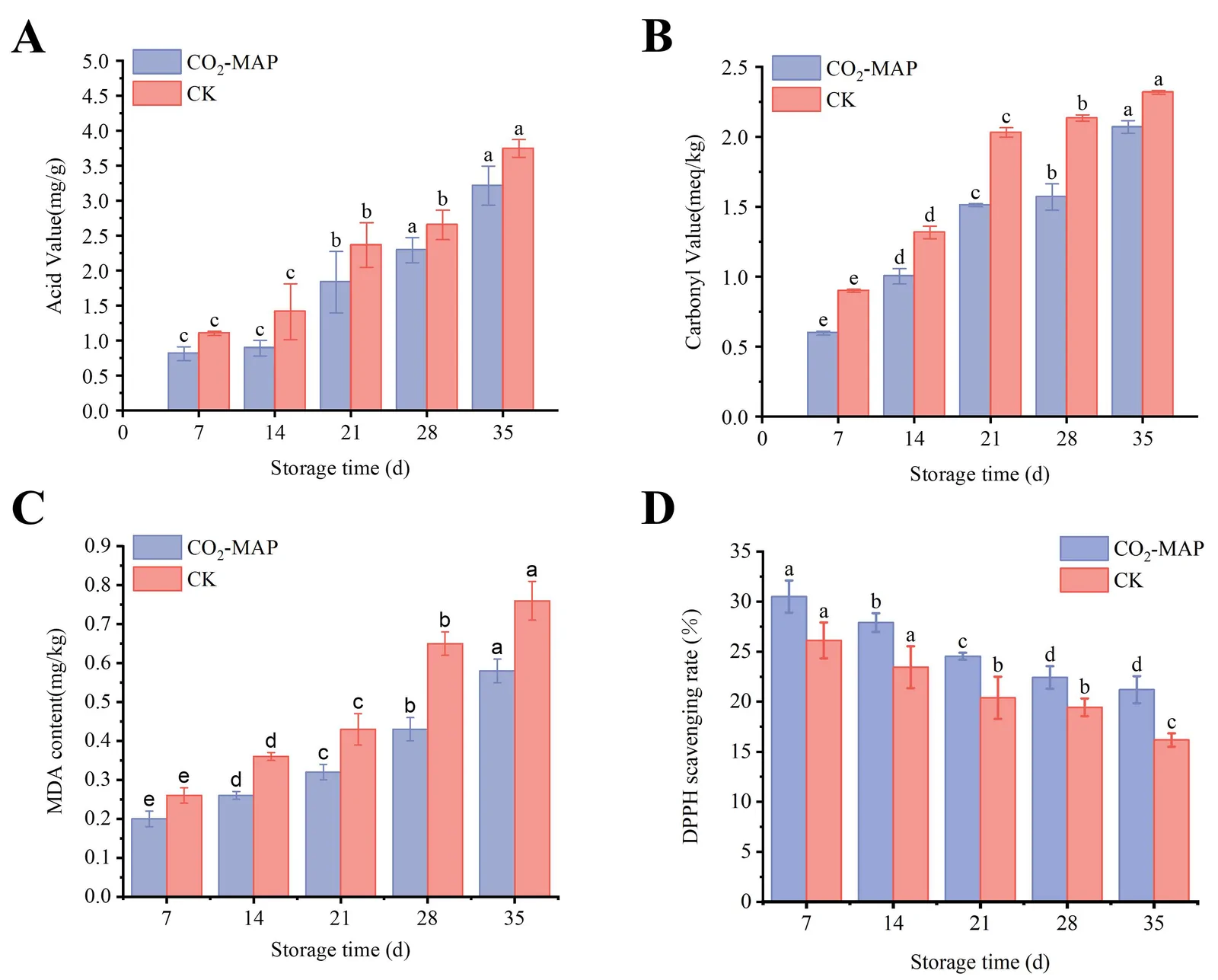
Effects of CO_2_-MAP on acid value (**A**), carbonyl Value (**B**), MDA content (**C**), and DPPH free radical scavenging rate (**D**) in fresh edible peanut kernels during storage. In the legend, CK = control, CO_2_-MAP = CO_2_ modified atmosphere packaging. Different lower-case letters indicated significant differences at different exposure times (Tukey test, α = 0.05).

The acid value of peanut kernels increased progressively with storage time in both groups. The increase rate in the CK group was higher, and the magnitude of difference between the two groups changed substantially over the storage period (Figure 2A). The acid value remained at low levels and rose slowly in both groups at the early storage stage (0–7 d). At 14–21 d, the acid value in both groups increased quickly, increasing from 1.42 to 2.37 mg/g in CK and from 0.90 to 1.84 mg/g in the CO_2_-MAP group. At 21–35 d, the acid values continued to increase in both groups, reaching 3.75 mg/g in the CK and 3.22 mg/g in the CO_2_-MAP by 35 d. At the end of storage, the acid value of the CK remained significantly higher than that of the CO_2_-MAP, indicating that CO_2_-MAP treatment effectively suppressed lipid hydrolytic deterioration in fresh edible peanut kernels.

The carbonyl value of peanut kernels increased continuously with extended storage in both groups, and the carbonyl value in CO_2_-MAP was significantly lower than that in CK, indicating that CO_2_-MAP treatment exerted a marked suppressive effect on oxidative deterioration in stored peanuts (Figure 2B). The carbonyl value in the CK rose from 0.90 (7 d) to 2.03 meq/kg (21 d), corresponding to a cumulative increase of 126%, while that in the CO_2_-MAP increased from 0.60 (7 d) to 1.51 meq/kg (21 d), with a cumulative increment of 153%. Although both groups exhibited relatively high oxidation rates, the CO_2_-MAP group consistently remained at a lower oxidative level, with a smaller absolute increase in carbonyl value compared with the control. From 21 d to 35 d, the growth rate of carbonyl value decelerated substantially in both groups, and the value reached 2.32 meq/kg in the CK and 2.07 meq/kg in the CO_2_-MAP at 35 d. The carbonyl value in the CO_2_-MAP group was 10.7% lower than that in the CK group, demonstrating an inhibitory effect on lipid oxidation.

The MDA content in peanut kernels increased continuously with storage time in both groups. Notably, the CO_2_-MAP group consistently maintained lower MDA levels (Figure 2C), indicating that CO_2_-MAP treatment effectively retarded lipid peroxidation and reduced the accumulation of oxidative products. MDA content was 0.20 mg/kg in the CO_2_-MAP and 0.26 mg/kg in the CK at 7 d. During the early storage phase (7–14 d), MDA contents remained at low levels and rose slowly in both groups, reaching 0.26 mg/kg in the CO_2_-MAP and 0.36 mg/kg in the CK at 14 d, respectively. After 14 d, the MDA accumulation accelerated markedly in both treatments, and the content reached 0.76 mg/kg in the CK group and 0.58 mg/kg in the CO_2_-MAP group at 35 d.

### 3.3. Changes of DPPH Free Radical Scavenging Activity

The DPPH free radical scavenging activity of peanut kernels declined continuously with storage time in both the CO_2_-MAP and CK, and values in CO_2_-MAP were consistently higher than those in the CK throughout the storage period (Figure 2D). The DPPH free radical scavenging rate decreased slowly during the early storage period. It dropped from 26.12% (7 d) to 23.45% (14 d) in the CK group, and from 30.50% (7 d) to 27.90% (14 d) in the CO_2_-MAP group. The decline rate accelerated after 14 d, and the activity fell to 20.39% in the CK group and 24.54% in the CO_2_-MAP group at 21 d. From 21 d to 35 d, the activity continued to decrease in both groups, with a more pronounced reduction observed in the CK group, particularly between 28–35 d. The DPPH scavenging activity dropped to 16.17% in the CK group, whereas it remained at 21.20% in the CO_2_-MAP group at 35 d. CO_2_-MAP treatment effectively retarded the loss of free radical scavenging capacity, which helped mitigate oxidative quality deterioration of peanuts during storage.

### 3.4. Changes of SOD and LOX Activity

**Figure 3:**
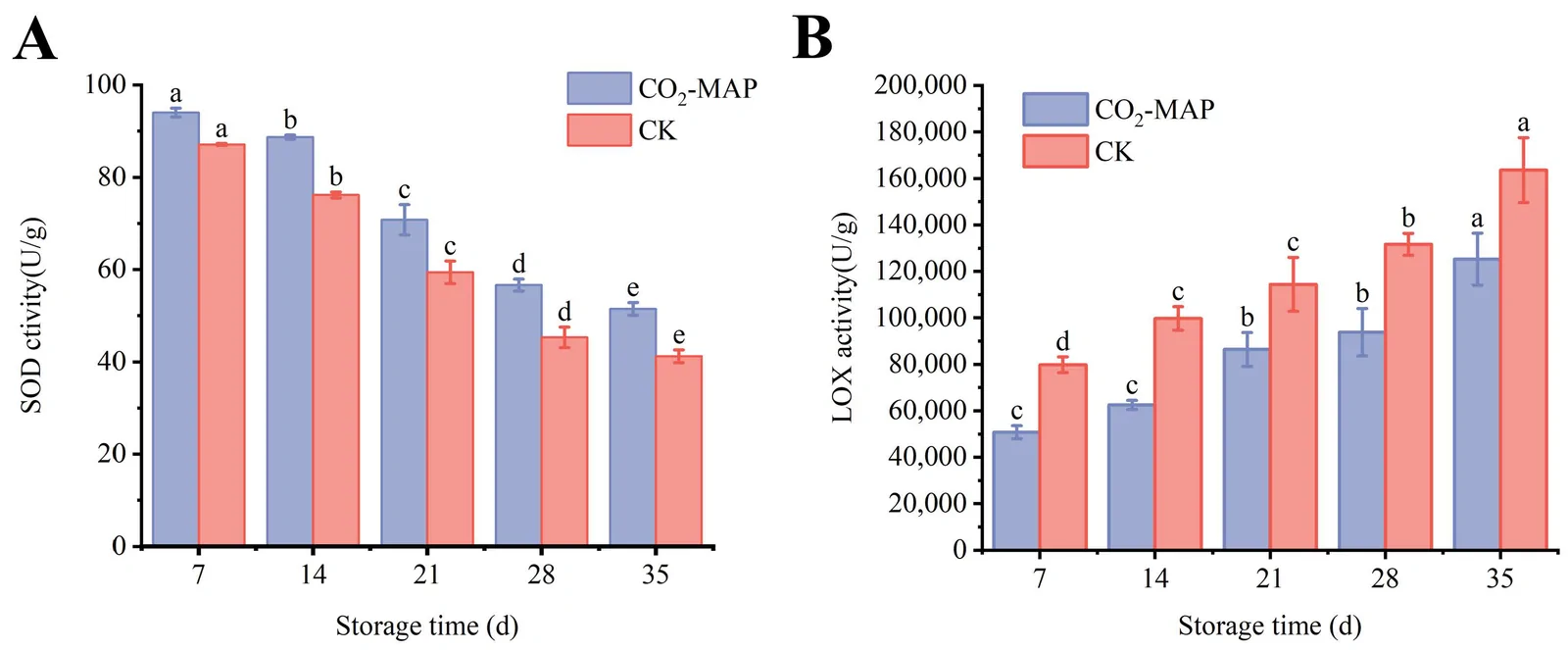
Effects of CO_2_-MAP on superoxide dismutase, SOD (**A**), and Lipoxygenase, LOX (**B**) activity of fresh edible peanut kernels during storage. In the legend, CK = control, CO_2_-MAP = CO_2_ modified atmosphere packaging. Different lower-case letters indicated significant differences at different exposure times (Tukey test, α = 0.05).

SOD activity in peanut kernels declined continuously throughout the storage period in both groups, and the activities in CO_2_-MAP were higher than those in the control (Figure 3A). SOD activity was 94.01 U/g in the CO_2_-MAP group and 87.10 U/g in the CK group at 7 d. A pronounced acceleration in SOD activity decline was observed in both groups between 14 and 21 d. Over this interval, SOD activity in the CO_2_-MAP group fell from 88.69 U/g to 70.78 U/g, and decreased from 76.16 U/g to 59.40 U/g in the CK group. The rate of SOD activity reduction moderated in both groups in 28–35 d. The CO_2_-MAP group declined from 56.65 U/g to 51.48 U/g, while the CK group declined from 45.31 U/g to 41.21 U/g. SOD activity in the CO_2_-MAP group was 25% higher than that in the CK group, confirming that CO_2_-MAP maintained a significant advantage in preserving the endogenous antioxidant defense system of peanuts during storage.

LOX activity in peanut kernels increased with storage time in both control and CO_2_-MAP groups (Figure 3B). LOX activity in the CO_2_-MAP group was lower than that in the CK, indicating that CO_2_-MAP treatment effectively suppressed the elevation of LOX activity in stored peanuts. Differences in LOX activity were already apparent at 7 d between the CK (79,800.00 U/g) and CO_2_-MAP group (50,800.00 U/g). By 14 d, LOX activity increased to 99,733.33 U/g in the CK group and 62,533.33 U/g in the CO_2_-MA group, respectively. From 21 to 28 d, LOX activity in the CK rose by 15% (from 114,433.30 U/g to 131,666.70 U/g), whereas the CO_2_-MAP group showed a more moderate increase of 9% (from 86,400 U/g to 93,800 U/g). At 35 d, LOX activity in the CO_2_-MAP group was 23% lower than that in the CK group, confirming that CO_2_-MAP maintained a significant suppressive effect on LOX activity throughout the entire storage period.

### 3.5. Transcriptome Sequencing Data Analysis

After quality filtering, a total of 39.76 Gb clean bases were obtained from samples. The total number of reads from the raw sequencing data of samples was at least 41.92 M and at most 48.96 M, respectively. The total number of reads after filtering was at least 40.61 M and at most 47.80 M, respectively (Table 1). The total number of bases in the raw sequencing data was at least 6.29 Gb and at most 7.34 Gb, respectively, and the total number of bases after filtering was at least 6.23 Gb and at most 7.15 Gb, respectively. The Q20 and Q30 values of all samples ranged from 95.85% to 96.60% and 93.08% to 94.29%, respectively, and the GC content ranged from 48.06% to 54.19. In addition, the effectiveness rate in each sample group was above 96%. These indicated that the sequencing quality satisfied the requirements for transcriptomic sequencing and could be accurately used for subsequent analyses. The sequencing data were submitted to the NCBI Database (BioProject accessions: PRJNA1502420).

### 3.6. Identification of Differentially Expressed mRNAs by RNA-Seq

**Figure 4:**
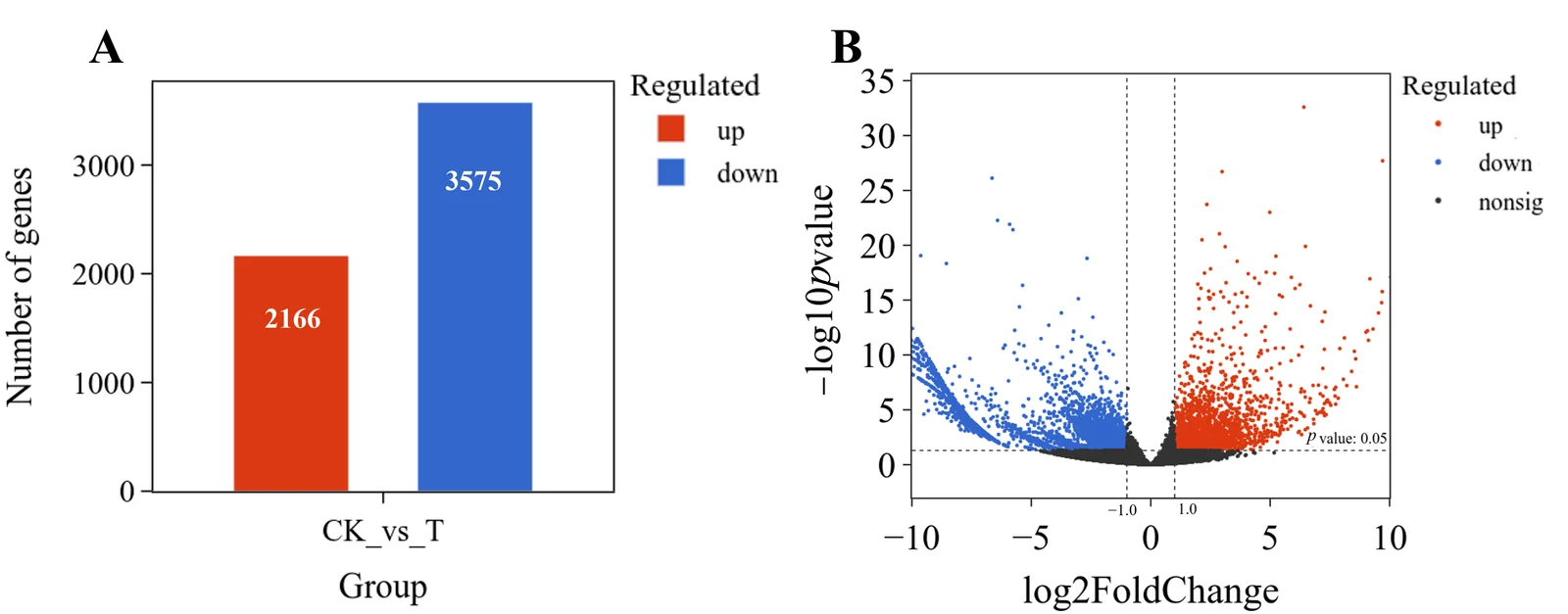
Identification of DEGs between CO_2_ modified atmosphere packaging and control. (**A**) The number of DEGs between CO_2_ modified atmosphere packaging and control. (**B**) The volcano plot of DEGs between CO_2_ modified atmosphere packaging and control. The *p*-value < 0.05 and |log2 fold change| ≥ 1 provided significance thresholds for gene expression differences. Of the 5741 identified DEGs, 2166 were upregulated, and 3575 were downregulated. Blue spot: downregulated DEGs; red spot: upregulated DEGs. Biological replicates (n = 3) for two conditions were performed. CK: control group; T: CO_2_ MAP treatment group.

DEGs between CO_2_ treatment and CK were determined by DESeq2, and the DEGs were identified by a *p*-value of <0.05 and |log2FC| ≥ 1. A total of 5741 DEGs were identified between the CO_2_ treatment and CK groups, including 2166 significantly upregulated and 3575 significantly downregulated genes (Figure 4). This indicated that peanut kernels underwent significant changes in their transcriptome when exposed to a CO_2_ atmosphere. The number of downregulated genes was notably higher than that of upregulated genes, indicating that CO_2_ treatment predominantly inhibited the expression of a large set of genes in stored peanut kernels.

**Figure 5:**
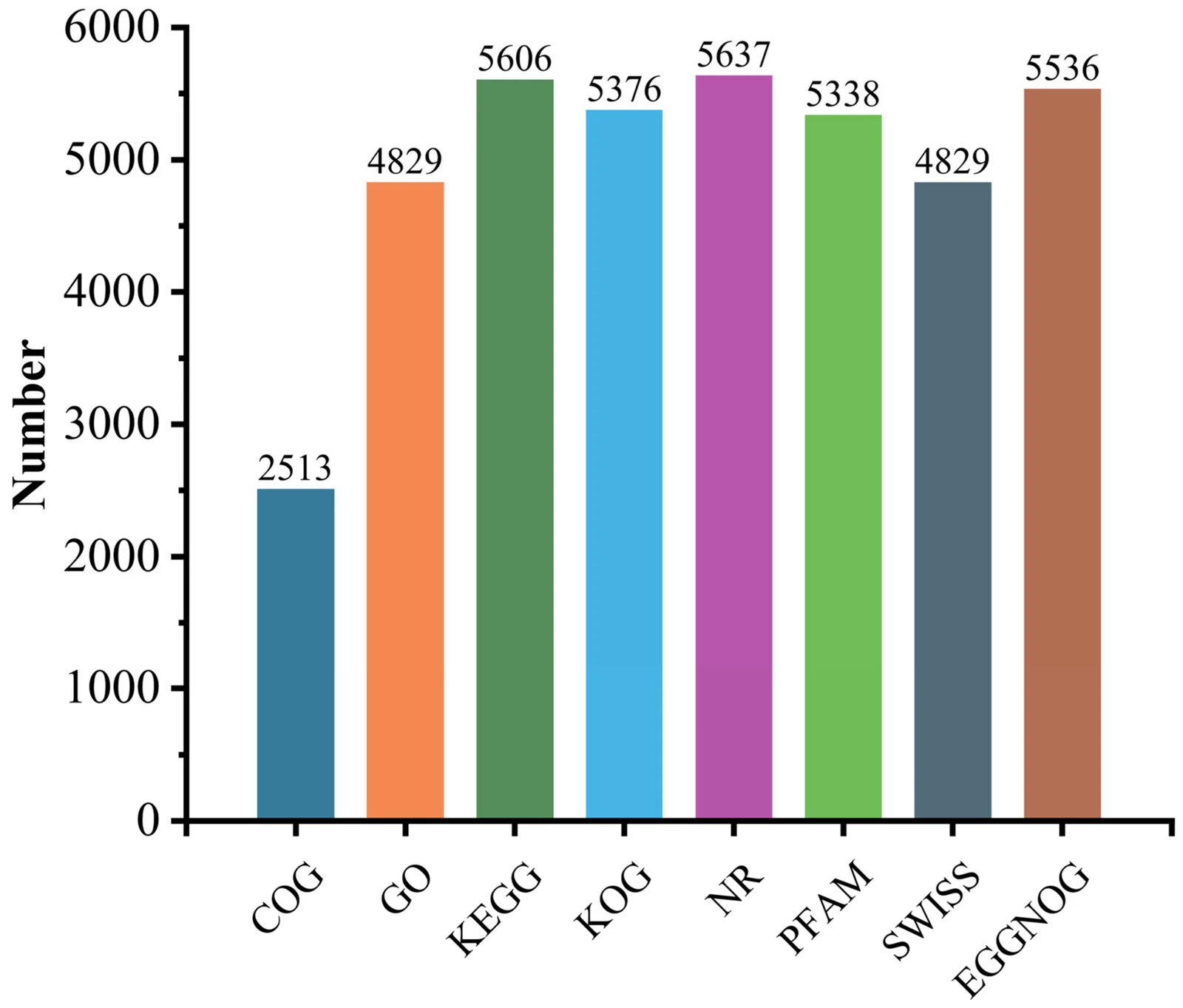
Statistics on the functional annotation of differentially expressed genes.

Functional annotation of DEGs was carried out with COG, GO, KEGG, KOG, NR, PFAM, SWISS, and EGGNOG databases, and the number of genes annotated in COG, GO, KEGG, KOG, NR, PFAM, SWISS, and EGGNOG was 2513 (43.77%), 4829 (84.11%), 5606 (97.65%), 5376 (93.64%), 5637 (98.19%), 5338 (92.98%), 4829 (84.11%), and 5536 (96.43%), respectively (Figure 5).

### 3.7. GO Enrichment Analysis of DEGs

**Figure 6:**
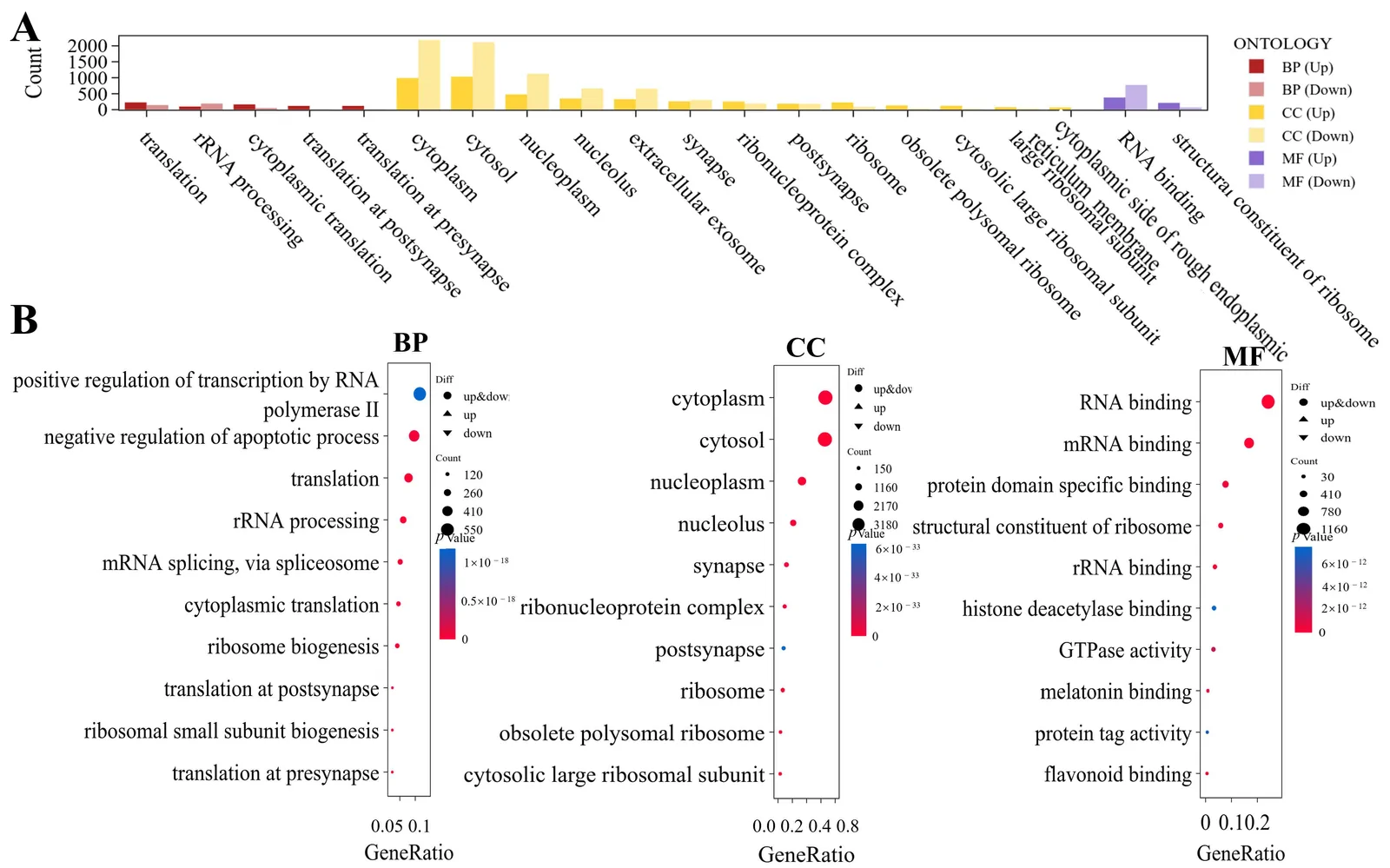
GO significant enrichment analysis of differentially expressed genes in peanut kernels under CO_2_-modified atmosphere packaging. (**A**). The significantly GO-enriched (Top 20 highest enrichment degree) analyses for total differentially expressed genes were shown in the bar chart. The horizontal axis represented pathway names, and the vertical axis indicated the number of enriched genes. The bar chart displayed the enriched terms and their degree of enrichment. (**B**). The enriched GO terms and the gene ratio were shown in the bubble plots. Gene Ontology was divided into three categories: Biological Process (BP), Cellular Component (CC), and Molecular Function (MF). The top 10 highest enrichment degrees of the three GO categories were shown in the bubble plots. The horizontal axis represented the proportion of DEGs within a specific category to the total number of genes in that category, while the vertical axis indicated different gene functional categories. The size of the circles reflected the number of genes enriched in each category, and larger circles indicated a greater number of genes enriched in that pathway. Color indicated enrichment significance, and the closer to red, the more significant.

GO enrichment analysis was performed to classify the biological functions (biological process BP, cellular component CC, and molecular function MF) of DEGs. The most significantly enriched terms in cellular component were cytosol (GO:0005829), nucleoplasm (GO:0005654), ribonucleoprotein complex (GO:1990904), obsolete polysomal ribosome (GO:0042788), ribosome (GO:0005840), and nucleolus (GO:0005730). In biological processes, cytoplasmic translation (GO:0002181), translation at presynapse (GO:0140236), and translation at postsynapse (GO:0140242) were the most enriched. In molecular function, structural constituent of ribosome (GO:0003735) was the most significantly enriched term. Based on the GO enrichment result, the top 20 highest enrichment degree of GO terms were listed in Figure 6A, and the top 10 highest enriched GO terms in three categories were listed in Figure 6B. These results indicated that CO_2_-MAP treatment mainly affected protein translation processes and ribosome-related functions in stored peanut.

### 3.8. KEGG Pathway Enrichment Analysis of DEGs

**Figure 7:**
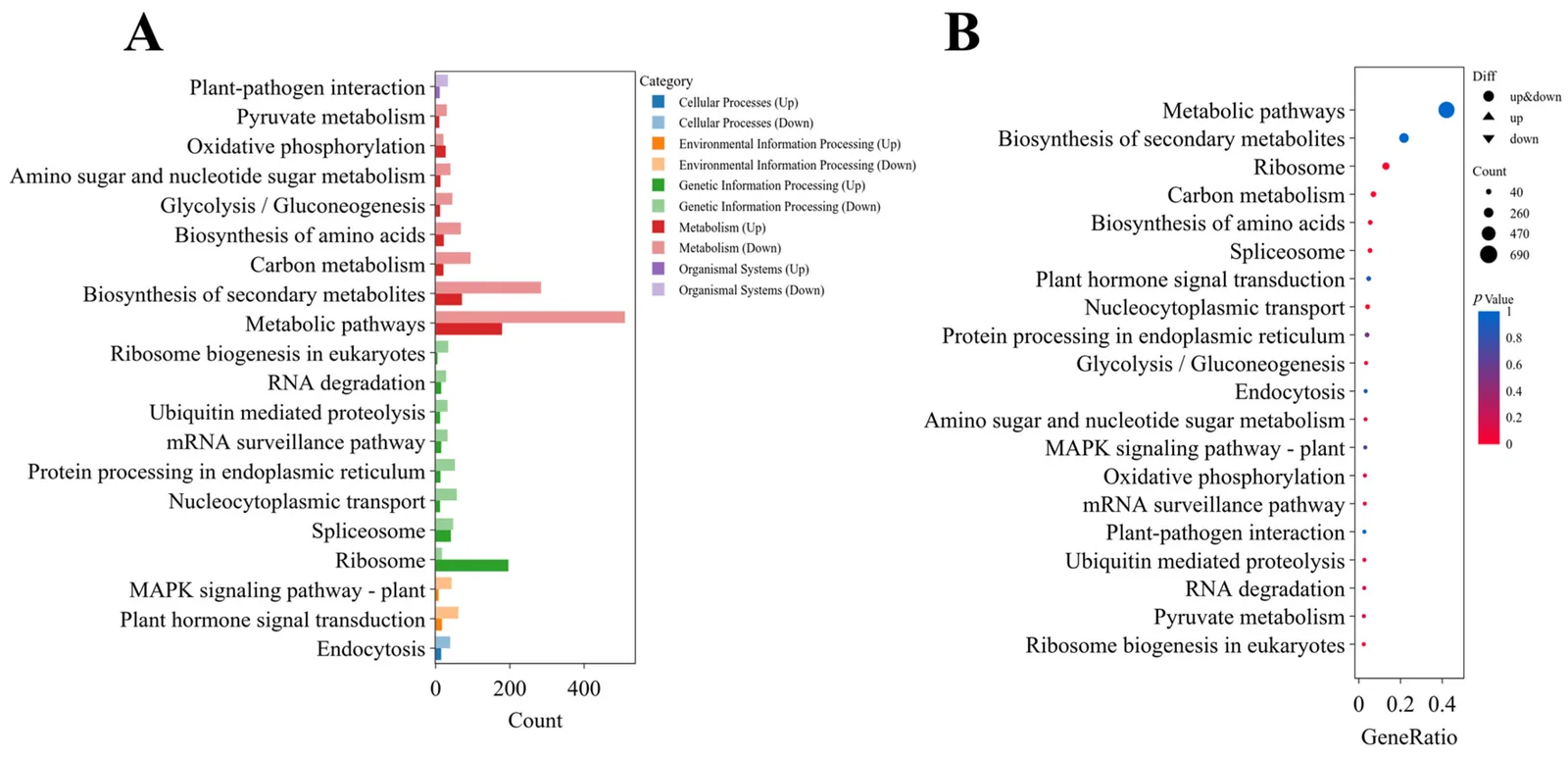
KEGG significant enrichment analysis of differentially expressed genes in peanut kernels under CO_2_-modified atmosphere packaging. (**A**) The enriched KEGG pathways (Top 20 most enriched) and the enriched DEG number of a specific KEGG pathway were shown in the bar chart. The horizontal axis represented the pathway names, and the vertical axis represented the number of DEGs enriched in this pathway. (**B**) The enriched KEGG pathways (Top 20 most enriched) and the gene ratio were shown in a bubble plot. The vertical axis indicated the pathway name, and the horizontal axis represented the gene ratio, which was the proportion of DEGs within a specific pathway to the total number of genes in that pathway. The size of the bubbles signified the number of gene enrichments, with color ranging from blue to red indicating increasing significance.

KEGG pathway enrichment was conducted to reveal metabolic and signal transduction pathways involved in CO_2_ response. Based on the KEGG pathway enrichment result, the top 20 most enriched pathways were listed in Figure 7. In the metabolism category, metabolic pathways (ko01100), biosynthesis of secondary metabolites (ko01110), carbon metabolism (ko01200), biosynthesis of amino acids(ko01230), glycolysis/gluconeogenesis (ko00010), fatty acid degradation (ko00071), and oxidative phosphorylation (ko00190) pathways were most enriched. In environmental information processing, plant hormone signal transduction (ko04075) and MAPK signaling pathway–plant (ko04016) pathways were the most enriched. In genetic information processing, ribosome (ko03010), spliceosome (ko03040), and nucleocytoplasmic transport (ko03013) were the most enriched. Additionally, plant-pathogen interaction (ko04626) was significantly enriched in the organismal systems category. These results suggested that CO_2_-MAP treatment could affect energy metabolism, carbon metabolism, lipid metabolism, signal transduction, and antioxidant functions in peanuts.

### 3.9. DEGs in Important Pathways

#### 3.9.1. DEGs in Oxidative Phosphorylation Pathway

**Figure 8:**
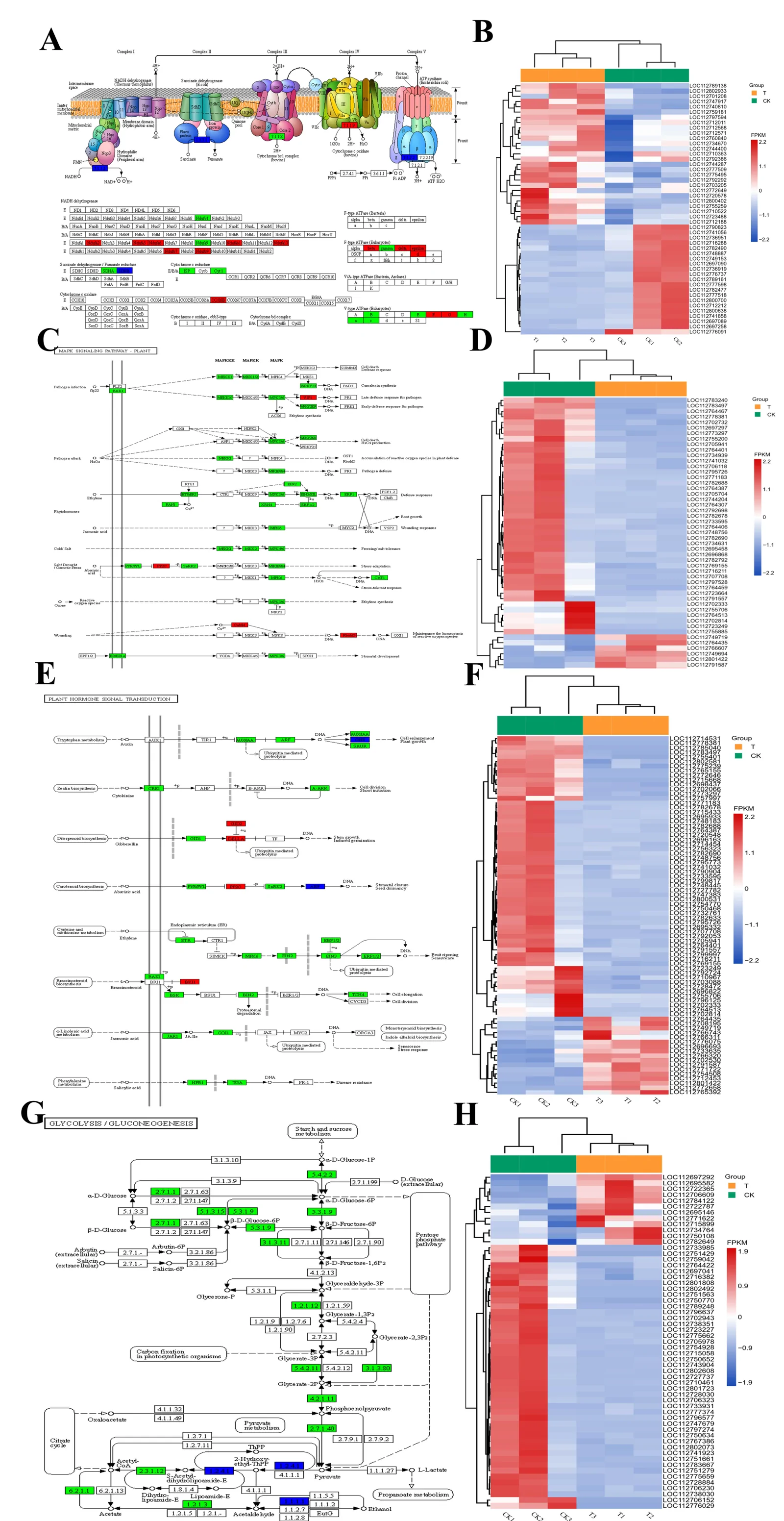
Heatmaps of differentially expressed genes in four important pathways and the pathway maps in peanut kernels under CO_2_-modified atmosphere packaging. (**A**) DEGs in oxidative phosphorylation pathway. (**C**) DEGs in the MAPK signaling pathway-plant pathway. (**E**) DEGs in the plant hormone signal transduction pathway. (**G**) DEGs in the glycolysis/gluconeogenesis pathway. Red and green frames represented the upregulated and downregulated genes, respectively; Genes that did not change were shown in black frames; blue frames represented inconsistently up- and downregulated. (**B**) Heatmap of DEGs in the phosphorylation pathway. (**D**) Heatmap of DEGs in the MAPK signaling pathway-plant pathway. (**F**) Heatmap of DEGs in plant hormone signal transduction pathway. (**H**) Heatmap of DEGs in the glycolysis/gluconeogenesis pathway. The rows represented genes, and the columns represented samples. Biological replicates (n = 3) for two conditions were performed. CK: control group; T: CO_2_ MAP treatment group. Red and blue colors represented increased and decreased identified genes, respectively.

As the core cascade governing mitochondrial energy supply, the oxidative phosphorylation pathway (ko00190) was further investigated in this study. A total of 48 DEGs were annotated in this pathway, indicating a substantial transcriptional reprogramming of the mitochondrial electron transport chain and ATP synthase complexes in the response of peanut kernels to CO_2_-MAP treatment (Figure 8A). After CO_2_-MAP treatment, 27 genes were significantly upregulated, and the remaining 21 genes were downregulated. As depicted in the pathway map and corresponding differential gene expression heatmap (Figure 8B), these DEGs encoded structural and catalytic subunits of the five respiratory complexes (Complex I–V) that drove electron transport and oxidative phosphorylation. NADH dehydrogenase showed mixed regulation, in which most DEGs of NDUF subunits were upregulated except for the *NDUFV1* and *NDUFA13* genes. DEGs encoding the cytochrome c reductase were uniformly downregulated, while genes encoding the cytochrome c oxidase were upregulated. Most subunits of complex V (ATP synthase) were downregulated, indicating remodeled ATP synthesis capacity.

#### 3.9.2. DEGs in MAPK Signaling Pathway—Plant Pathway

The plant MAPK signaling pathway (ko04016), a central cascade governing stress perception, oxidative stress response, defense activation, and hormone signal transduction, was markedly reshaped at the transcriptional level by CO_2_-MAP in peanut kernels (Figure 8C). As shown in Figure 8D, 51 DEGs were annotated to this pathway. Among these, only 8 DEGs were significantly upregulated, whereas 43 DEGs exhibited downregulated expression. DEGs in reactive oxygen species (*MPK3*/*MPK6*) and ethylene pathway components (*ETR*/*ERS*, *EIN3*/*EIL*, and *ERF1*/*2*) were downregulated, indicating suppression of the accumulation of reactive oxygen species (ROS) and ethylene-mediated senescence in peanut kernels with CO_2_-MAP treatment. DEGs related to pathogen attack, stomatal development, and cold/salt tolerance also exhibited global downregulation, implying attenuated abiotic stress signal transduction. DEGs associated with wounding response were upregulated, reflecting selective adaptive adjustment. Overall, the predominant downregulation of MAPK cascade genes indicated that CO_2_-MAP repressed accumulation of ROS, stress-induced signal transduction, excessive defense activation, and senescence progression, thereby contributing to quality maintenance in postharvest peanut kernels.

#### 3.9.3. DEGs in Plant Hormone Signal Transduction Pathway

The plant hormone signal transduction pathway, a central regulatory network governing postharvest senescence and stress adaptation, was transcriptionally modulated by CO_2_-MAP in peanut kernels. DEGs were annotated to this pathway, with 17 DEGs significantly upregulated and 61 DEGs downregulated after CO_2_ treatment (Figure 8F), reflecting an overall repressive effect on most hormone signaling cascades. DEGs in cytokinin (*CRE1*, *A-ARR*), ethylene signaling (*ETR*, *MPK6*, *CTR1*, *EIN2*, *EIN3*, *ERF1*/*2*), jasmonic acid (*JAR1*, *COI1*), and salicylic acid (*NPR1*, *TGA*) signaling showed global repression (Figure 8E), indicating suppressed cell division, ripening, and senescence signals. DEGs in auxin and brassinosteroid were predominantly downregulated, indicating suppressed cell enlargement and division activities during storage. In contrast, a small subset of genes involved in abscisic acid and gibberellin signaling were selectively upregulated, which may function to sustain basal defense capacity against stresses.

#### 3.9.4. DEGs in Glycolysis/Gluconeogenesis Pathway

A total of 57 DEGs were annotated to the glycolysis/gluconeogenesis pathway, with 12 genes significantly upregulated and 45 genes downregulated under CO_2_-MAP treatment (Figure 8H). Key rate-limiting enzyme genes involved in glycolysis, including hexokinase (*HK*, EC 2.7.1.1), phosphofructokinase (*PFK*, EC 2.7.1.11), and pyruvate kinase (*PK*, EC 2.7.1.40), were downregulated, indicating suppressed glucose catabolism and reduced pyruvate generation. In contrast, alcohol dehydrogenase (NADP+) (*AKR1A1*, EC 1.1.1.2) was upregulated (Figure 8G), likely maintaining basal glucose homeostasis. Overall, the widespread downregulation of glycolytic genes suggested that CO_2_-MAP attenuated cellular carbon catabolism, reduced energy consumption, and slowed respiratory turnover, thereby delaying senescence and preserving quality in postharvest peanut kernels.

## 4. Discussion

Postharvest quality loss of fresh edible peanuts severely limited their storage life and market value. The preservation effect of MAP storage largely depends on the precise regulation of O_2_ and CO_2_ concentrations. The present study demonstrated that CO_2_-MAP effectively mitigated appearance deterioration and lipid oxidative damage in fresh peanuts during 35 d of storage. Integrated physiological and transcriptomic analyses further revealed that the preservative effect of CO_2_-MAP was mediated by the coordinated regulation of energy metabolism, lipid peroxidation, antioxidant defense, stress signaling, and hormone transduction pathways.

### 4.1. Postharvest Quality of Peanuts

Color is a vital indicator for the quality of postharvest agricultural products, which directly affects consumer acceptance and commercial value [35]. In the present study, CO_2_-MAP markedly maintained the color and inhibited mold colonization compared with the control, and this effect became increasingly prominent with extended storage time. These findings agreed with previous reports that peanut kernels maintained an acceptable color in CO_2_-MAP [26] and N_2_-MAP treatment [25]. The improved appearance retention was closely coupled with the suppression of internal lipid oxidative deterioration. Also, CO_2_ could have been adsorbed into the pores of the peanut forming vacuoles inside pouches and dissolved in peanut cells, reducing pH and maintaining the color by reducing the browning reaction [26].

Acid value, carbonyl value, and MDA content are well-established indicators reflecting the extent of lipid hydrolytic rancidity and oxidative degradation [32,36,37]. In the present study, all three indices increased progressively over storage in both groups, but were consistently lower in the CO_2_-MAP group, indicating that CO_2_-MAP treatment effectively retarded both hydrolytic and oxidative lipid deterioration. Compared with the control, CO_2_-MAP maintained a lower acid value (14.3% lower), carbonyl value (10.7% lower), and malondialdehyde content (22.5% lower) at 35 d, respectively. Similarly, fresh edible peanut kernels under N_2_-MAP treatment consistently exhibited lower malondialdehyde content compared with the control. On 120 d, the value in N_2_-MAP-treated fresh edible peanut kernels stored at 4 °C was 9.3% lower than that in the control [25]. These results collectively indicated that the low-oxygen environment established by MAP effectively protected fresh peanut kernels from oxidative damage during storage. Such an effect was in agreement with previous findings reported in litchi fruit [38], cut peonies [19], and papaya fruit [39], indicating that MAP treatments could suppress lipid peroxidation and preserve membrane integrity during postharvest storage.

### 4.2. Antioxidant Capacity and Enzyme Activity

The antioxidant system, comprising both enzymatic and non-enzymatic components, serves as the primary defense against ROS and oxidative damage in postharvest crops. DPPH radical scavenging activity reflects overall non-enzymatic antioxidant capacity [40], while SOD constitutes the first line of enzymatic defense and catalyzes O_2_^−^ disproportionation to produce O_2_ and H_2_O_2_ to prevent oxidative damage [41,42,43]. In this study, both DPPH scavenging activity and SOD activity declined continuously during storage, but CO_2_-MAP significantly slowed this decline. This result indicated that CO_2_-MAP preserved the activity of antioxidant enzymes and reduced the consumption of antioxidant compounds, thereby sustaining a higher ROS scavenging capacity. Our findings were consistent with previous reports that the SOD activity in N_2_-MAP-treated fresh edible peanut kernels stored at 4 °C was significantly higher than that in the control after 40 d of storage [25].

LOX is an enzyme closely related to biotic and abiotic stress resistance in plants and catalyzes the oxidation of polyunsaturated fatty acids [44]. In our study, LOX activity was already significantly lower in the CO_2_-MAP group at 7 d, and the intergroup difference widened throughout storage. This inhibition of LOX activity suggested that CO_2_ may directly suppress enzyme activity or downregulate its transcriptional expression, thereby blocking the enzymatic lipid oxidation cascade. The reduction in LOX activity consequently limited the generation of reactive aldehydes and MDA, which was consistent with the lower carbonyl value and MDA content observed in CO_2_-MAP. Corresponding reductions in LOX have also been reported in peanuts under N_2_-MAP storage [45], where suppressed LOX was identified as a key mechanism for less oxidative stress.

The preserved antioxidant defense, combined with suppressed LOX activity, collectively contributed to reduced lipid peroxidation and quality maintenance in CO_2_-MAP peanuts. These results indicated that CO_2_-MAP treatment may enhance the antioxidant defense capacity and stress tolerance of fresh edible peanut kernels. Therefore, CO_2_-MAP treatments can protect them against oxidative damage, retard postharvest senescence, and preserve desirable commercial quality attributes.

### 4.3. Transcriptomic Insights into the Preservation Mechanisms of CO_2_-MAP

To elucidate the molecular basis underlying the preservative effect of CO_2_-MAP, we performed comparative transcriptome profiling between CO_2_-MAP and control peanut kernels. A total of 5741 DEGs were identified, with downregulated DEGs (3575) outnumbering upregulated DEGs (2166). These DEGs were significantly enriched in pathways related to energy metabolism, carbon metabolism, lipid metabolism, signal transduction, and antioxidant defense in peanuts treated by CO_2_-MAP, indicating that CO_2_-MAP primarily exerted its regulatory role by repressing genes associated with energy metabolism, catabolic metabolism, and stress response.

### 4.4. Reprogramming of Energy Metabolism Pathways

Glycolysis and oxidative phosphorylation are central pathways governing cellular energy supply and respiratory metabolism, and their activity is tightly linked to the quality attributes in postharvest commodities [46]. In the present study, key rate-limiting enzymes of glycolysis (hexokinase, phosphofructokinase and pyruvate kinase) were significantly downregulated at the transcriptional level under CO_2_-MAP. The global downregulation of glycolytic genes suggested that a CO_2_ atmosphere suppressed glucose catabolism and reduced pyruvate generation, which in turn decreased respiratory intensity and energy consumption. In agreement with our results, Qu et al. [47] reported that nitrogen-controlled atmosphere could regulate carbohydrate metabolism to delay rice deterioration, and Li et al. suggested that nitrogen atmosphere could remodel energy metabolism and redox homeostasis in peanuts [45].

In the oxidative phosphorylation pathway, most subunits of ATP synthase (Complex V) were downregulated, consistent with the reduced energy demand under suppressed respiratory metabolism. Similarly, the CA group maintained a high phosphorylation level of cut peony after 15 d of storage, while the CK group almost lost its phosphorylated state [19]. Notably, genes encoding cytochrome c oxidase (Complex IV) in our study were upregulated, whereas cytochrome c reductase (Complex III) subunits were uniformly downregulated. Such bidirectional transcriptional regulation indicated that CO_2_ treatment remodeled respiratory chain function. It may reduce overall respiration intensity and electron leakage-derived ROS production, while retaining basal ATP synthesis capacity, which contributed to delayed senescence and quality maintenance in postharvest peanut kernels. Excessive ROS generated from mitochondrial electron leakage can cause lipid peroxidation, protein oxidation, and enzyme inactivation [48]. The imbalance of ROS metabolism in plants accelerated the senescence of plant tissue or lipid peroxidation [49]. Therefore, the remodeling of oxidative phosphorylation by CO_2_-MAP may represent an important mechanism for reducing oxidative damage and membrane damage in stored peanut kernels.

### 4.5. Attenuation of Stress Signaling and Senescence-Related Hormone Pathways

MAPK pathway is a conserved signaling module that mediates stress perception, ROS signal amplification, and senescence initiation in plants [50]. In our study, the majority of DEGs in the plant MAPK signaling pathway were downregulated under CO_2_-MAP, including core components involved in ROS response and ethylene signaling. The downregulation of *MPK3*/*MPK6* indicated that a CO_2_ atmosphere suppressed ROS-mediated stress signal transduction, which was consistent with the reduced MDA content and enhanced antioxidant capacity observed at the physiological level. Collectively, the widespread transcriptional repression of the MAPK cascade suggested that CO_2_-MAP alleviated stress-triggered signal transduction, reduced defense-related energy expenditure, and delayed senescence progression, thereby preserving postharvest quality of peanuts.

Furthermore, the global downregulation of ethylene signaling components (*ETR*, *EIN3*, *ERF1*/*2*) in both the MAPK and plant hormone signal transduction pathways suggested that CO_2_-MAP inhibited ethylene biosynthesis and signal transduction. Ethylene is a gaseous hormone that plays important roles in both plant growth and development and stress responses. It includes ethylene-induced NAC transcription factor functions regulating ripening [51,52]. Our results showed that this was one of the key mechanisms of quality preservation in peanut under CO_2_-MAP, indicating that suppressing ethylene signaling was recognized as an effective strategy for delaying lipid oxidation and quality deterioration in postharvest produce.

Consistently, the plant hormone signal transduction pathway showed overall transcriptional repression under CO_2_-MAP. Beyond ethylene signaling, genes involved in jasmonic acid, salicylic acid, and cytokinin signaling were largely downregulated, which was consistent with the lower LOX activity in the CO_2_-MAP group. Jasmonic acid and salicylic acid are defense-related hormones, which can exacerbate oxidative stress when hyperactivated [53,54]. The repression of these defense pathways likely reflected the reduced stress status of CO_2_-treated peanuts, as the high CO_2_ environment already mitigated microbial pressure and oxidative damage. A small subset of genes involved in abscisic acid and gibberellin signaling were selectively upregulated, which may function to maintain basal stress tolerance and cellular homeostasis during storage. Taken together, these transcriptomic changes demonstrate that CO_2_-MAP modulated a complex hormone signaling network to delay senescence and preserve quality in postharvest peanuts.

## 5. Conclusions

CO_2_-MAP effectively maintained the color, inhibited mold growth and lipid oxidative deterioration during 35 d storage, as reflected by lower acid value, carbonyl value, and MDA content. Meanwhile, CO_2_-MAP suppressed LOX activity and preserved higher DPPH radical scavenging capacity and SOD activity, thereby sustaining endogenous antioxidant defense. Transcriptomic analysis identified 5741 DEGs between the two groups, which were mainly enriched in carbon metabolism, lipid metabolism, oxidative phosphorylation, signal transduction, and antioxidant functions. The number of downregulated genes was notably higher than that of upregulated genes, indicating that CO_2_-MAP mainly exerted its regulatory role by repressing genes associated with catabolic metabolism, stress response, and senescence. In conclusion, CO_2_-MAP could delay the quality deterioration of peanuts by suppressing lipid oxidation, enhancing antioxidant capacity, reprogramming energy metabolism and attenuating senescence-associated signaling pathways.

## Figures and Tables

**Table 1:** Summary of sequence data for peanut kernels in response to CO_2_ modified atmosphere packaging.

Sample	Raw Reads (M)	Raw Bases (G)	Clean Reads (M)	Clean Bases (G)	Effective Rate (%)	GC (%)	Q20 (%)	Q30 (%)
CK1	48.18	7.23	46.82	7.01	96.96	52.89	96.24	93.70
CK2	46.76	7.01	45.53	6.82	97.21	48.06	96.25	93.69
CK3	41.92	6.29	40.61	6.08	96.72	54.19	95.85	93.08
T1	48.96	7.34	47.80	7.15	97.37	49.68	96.60	94.29
T2	44.21	6.63	43.26	6.48	97.64	49.35	96.49	94.12
T3	42.60	6.39	41.63	6.23	97.50	49.33	96.51	94.15

CK: control group; T: CO_2_ MAP treatment. Raw Reads: Total number of reads from the raw sequencing data; Raw Bases: Total number of bases in the raw sequencing data; Clean Reads: Total number of reads after filtering; Clean Bases: Total number of bases after filtering; Effective Rate: Data effectiveness rate (Effective Rate = Clean Bases/Raw Bases); GC: GC content, the percentage of G and C bases out of the total number of bases; Q20: the percentage of bases with a Phred score greater than 20 out of the total number of bases; Q30: the percentage of bases with a Phred score greater than 30 out of the total number of bases.

## Data Availability

The original contributions presented in the study are included in the article, further inquiries can be directed to the corresponding authors.
